# Seroprevalence and Risk Factors of Sexually Transmitted Blood-Borne Infections among Pregnant Women Attending Antenatal Care in Jirapa, Upper West Region of Ghana

**DOI:** 10.1155/2023/3157202

**Published:** 2023-04-30

**Authors:** Eugene D. Kuugbee, Gloria Maaldu, Aseta Adamu, Nafisa Salia, Williams Walana, Sylvanus Kampo, Ezekiel K. Vicar, Juventus B. Ziem

**Affiliations:** ^1^School of Medical Sciences, C. K. Tedam University of Technology and Applied Sciences, Navrongo, UER, Ghana; ^2^School of Nursing and Midwifery, University for Development Studies, Tamale, Ghana; ^3^Obstetrics and Gynecology Unit, St Joseph's Hospital, P.O. Box 3, Jirapa, UWR, Ghana; ^4^Department of Clinical Microbiology, School of Medicine, University for Development Studies, Tamale, Ghana

## Abstract

**Background:**

Sexually transmitted blood-borne infections (STBBIs) contribute to negative outcomes of pregnancy. Hepatitis B virus (HBV), hepatitis C virus (HCV), human immunodeficiency virus (HIV), and syphilis infections in pregnancy contribute significantly to maternal and child morbidities and mortalities. This study assessed the prevalence, knowledge, and risk factors of STBBIs (HBV, HCV, HIV, and syphilis) among pregnant women attending antenatal clinics in Jirapa.

**Methods:**

A cross-sectional study design involving 246 pregnant women was employed for the study. A structured questionnaire was used to solicit information about the knowledge, prevalence, and risk factors of STBBIs.

**Results:**

The overall prevalence of STBBIs was 11.4%; HBV prevalence was 9.8% and 0.8% each for HCV, HIV, and syphilis. About 66% of mothers were aware of mother-to-child transmission of infections during pregnancy. Knowledge of transmission of HIV (93.9%), hepatitis (67.1%), and syphilis (53.7%) in pregnancy was relatively high. Knowledge of risk factors for HIV, hepatitis, and syphilis was 97.6%, 74.4%, and 76.0%, respectively. More than 98% of respondents knew about the prevention of HIV, hepatitis, and syphilis. Significant risk factors associated with and predictive of STBBIs were female genital mutilation (FGM) and gravidity.

**Conclusion:**

The occurrence of STBBIs among pregnant women was strongly associated with FGM and gravidity. Public health education should be directed at stopping the practice of FGM and improving reproductive health in the study area.

## 1. Introduction

Pregnancy is usually accompanied by immunologic changes which induce a state of predisposition and susceptibility to various forms of infections [1] and could increase maternal anxiety and uncertainty about pregnancy outcomes [2].

Sexually transmitted blood-borne infections (STBBIs), including human immunodeficiency virus (HIV), syphilis, hepatitis B virus (HBV), and hepatitis C virus (HCV), are infections transmitted during sexual contact via blood and or other body fluids. In developing countries, infectious disease prevalence is relatively high, and STBBIs are particularly important in determining the outcome of pregnancies [3].

In 2016, the World Health Organization (WHO) approximated 376 million new sexually transmitted infections with one of four of the following infections: trichomoniasis (156 million), chlamydia (127 million), gonorrhea (87 million), and syphilis (6.3 million). Currently, sexually transmitted infections acquired daily are estimated at more than 1 million. Also, approximately 240 million people globally are living with chronic hepatitis B although hepatitis B infection is vaccine-preventable [4].

Sexually transmitted and blood-borne infection prevention and control have widespread public health benefits and contribute to progress toward the sustainable development goals. To reduce pregnancy-related complications and improve pregnancy outcomes, the WHO recommended Focused Antenatal Care, which includes routine screening for STBBIs during antenatal visits [5, 6]. Complications resulting from STBBIs are among the major causes of maternal and neonatal morbidity and mortality during pregnancy [7].

Many women and adolescents lack knowledge about STBBIs and relevant services, including prevention and management [8].

STBBIs in pregnancy are particularly associated with detrimental aftereffects to both the baby and the mother [5] and may lead to spontaneous abortion, preterm birth, fetal infection, and psychosocial stress on the mother [9].

In this study, authors assessed the knowledge and prevalence of STBBIs (syphilis, HBV, HCV, and HIV) and associated risk factors in pregnancy.

## 2. Materials and Methods

### 2.1. Study Setting and Subjects

This cross-sectional study targeted pregnant women attending antenatal clinic at the St. Joseph Hospital in the Jirapa municipality. The hospital is the only referral hospital in the Jirapa municipality, located in Jirapa in the upper west region, Ghana. The hospital has the following units through which it provides services to patients: pediatric, surgical, medical, obstetric, physiotherapy, outpatients' department and diagnostic (radiology and laboratory services). The antenatal services are provided by the obstetric unit of the hospital.

### 2.2. Sample Size Estimation

The sample size was estimated using Yamane's formula [10]:

(1) $$\begin{array}{c} n=\frac{N}{1+N*{\left(e\right)}^{2}}\text{,} \end{array}$$

where *n* = the desired sample size, *N* = the population size estimated at 590 from the antenatal attendance records, and *e* = acceptable sample size error assumed at 5% (level of precision) at a confidence level of 95%. This gave a minimum sample size of 238 participants for the study. However, a sample of 246 was achieved.

### 2.3. Data Collection

A structured questionnaire was designed with some modifications [11] and used to obtain information from respondents from August to November 2019. The data obtained included respondents' sociodemographic characteristics, obstetric characteristics, knowledge, and risk factors of STBBIs in pregnancy.

The respondents' status of HBV, HCV, HIV, and syphilis was obtained from the laboratory analysis of 5 mls of blood obtained from each participating pregnant woman into an EDTA tube. These tests are part of the Ghana Health Service's mandatory screening protocols for antenatal attendees. Rapid diagnostic test cassettes (Innovita Biological Technology Co. Ltd., China) were used for the screening for hepatitis B surface antigen (HBsAg), anti-HCV, and syphilis according to manufacturer protocols. HIV screening was according to the national rapid testing algorithm using HIV first response test kit and Ora quick (MoH, Ghana). All positive tests for HIV were confirmed using ELISA.

### 2.4. Data Analysis

The data were entered in Microsoft Excel version 2016. This was later exported to the Statistical Package for Social Sciences (SPSS) version 25 for analysis. Categorical variables were presented as proportions. Continuous variables were presented as means with standard deviation. The knowledge score was calculated from 13 structured questions on STBBIs in pregnancy, preventive practices, signs and symptoms, and transmission. A correct answer was awarded 1 point. An overall score below the 50^th^ percentile was classified as poor, between the 50^th^ and the 75^th^ percentile as moderate, and above the 75^th^ percentile as good knowledge. The continuous variables, gravidity, and parity were classified. Gravidity (number of pregnancies) was classified into two groups: primigravida (1 pregnancy), and multigravida (2 or more pregnancies). Also, parity (number of births) was classified into three groups: nullipara (no birth), primipara (1 birth), and multipara (2 or more births).

The chi-square test was used to compare proportions. To determine the associated risk factors of STBBIs in pregnancy, we performed univariate logistic regression analysis. A risk factor was significant at *p* < 0.05. All variables that met the assumptions for analysis in univariate analysis were further analyzed in a multiple logistic model. For the purpose of the analysis, the presence of STBBIs was calculated as being positive for either HBV, HCV, HIV, or syphilis. Other categorical responses to some risk factors were categorized as “Yes” or “No” and include a family history of HBV, HCV, and HIV infection. Education was reclassified as low (having at most primary education) and high (having at least high school education). The profession was categorized as “Salaried” and “Nonsalaried,” and “Age” as ≤mean (26) and >mean (26).

### 2.5. Ethical Statement

The study was approved by the ethics review board of Tamale Teaching Hospital (TTH/R&D/SR/151) and followed the ethical guidelines of St Joseph's Hospital. The aim and purpose of the study were explained to all respondents. A written informed consent was obtained from respondents to participate in the study.

## 3. Results

### 3.1. Respondents' Sociodemographic Characteristics

A total of 246 respondents were included in the study. Their ages ranged from 13.0 to 45.0 years with a mean age of 26.3 ± 6.2 years. Christianity constituted the dominant religion of the respondents (87.8%) while 85.4% of the respondents were married. The literacy rate was 79.9% with the educated having a variable level of education: tertiary (16.7%), secondary (17.0%), and primary (47.2%). Despite this, only 16.3% of the respondents were engaged in formal employment as shown in Table 1.

Among the respondents, 57.2% had body scarifications while 23.2% of them had female genital mutilation (FGM). History of blood transfusion, abortion, and other STBBIs were recorded in 10.2%, 13.8%, and 6.1% of the respondents, respectively. Overall, 13.8% of the respondents confirmed they have been vaccinated against HBV (Table 1).

With respect to the obstetrics history, the gravidity of the respondents ranged from 1 to 8 pregnancies with a mean gravidity of 2.4 ± 1.5 pregnancies. While 36.6% of the respondents were primigravidae, 63.4% were multigravida with 8.9% being grand multigravida. The parity of the respondents ranged from 0 to 7 live births with a mean parity of 1.2 ± 1.4. In all, 39.9% were nulliparous, 26.8% were primiparous, and 33.8% were multiparous with 4.1% being grand multiparous (Table 1).

### 3.2. Respondents' Knowledge of Sexually Transmitted Diseases and Risk Factors

In all, greater than half (50%) of the pregnant women knew the clinical signs and presentation, prevention, transmission, and risk factors of HBV, HCV, HIV/AIDS, and syphilis as shown in Table 2. The overall knowledge level of the respondents was scored from 7 to 13. The mean score was 11.0 ± 1.7. Based on the knowledge level classification, the knowledge rating was good in 81.7% (201) of the respondents and moderate in 18.3% (45), and none had a poor knowledge level.

### 3.3. Prevalence of Sexually Transmitted Blood-Borne Infections among Participants

**Figure 1:**
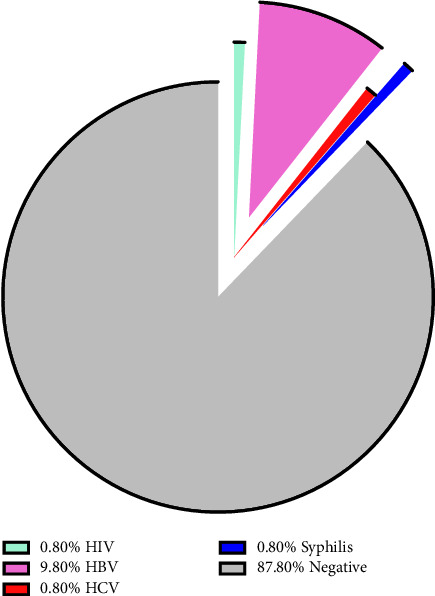
Prevalence of STBBIs among pregnant women attending antenatal clinic in Jirapa.

In all, 11.4% of the respondents were infected with one or more of the STBBIs investigated. Specifically, the prevalence of the studied infections was 9.8% for HBV and 0.8% each for HCV, HIV, and syphilis as shown in Figure 1. HBV coinfection with syphilis 1(0.4%) and with HCV 1(0.4%) was observed (data not shown).

### 3.4. Sexually Transmitted Blood-Borne Infections Associated Risk and Predictive Factors

Univariate logistic regression analysis revealed female genital mutilation (FGM) and gravidity were significantly associated with the occurrence of STBBIs studied. Knowledge of STBBIs was not associated with the occurrence of STBBIs (Table 3). In the multiple logistic regression analysis, FGM (OR *=* 2.88, 95% CI *=* 1.3–6.5, *p*=0.01) and gravidity (OR *=* 2.19, 95% CI *=* 1.1–7.9, *p*=0.04) significantly influenced the occurrence of STBBIs. The odds of a pregnant woman with FGM with STBBIs increased by 1.88 times compared with a pregnant woman without FGM. Also, the odds of a multigravida compared with a primigravida pregnant woman with STBBIs increased by 1.19 times as shown in Table 3.

We further stratified the various STBBIs studied to investigate their influencing factors among pregnant women. Only HBV infection met the criteria for analysis as shown in Table 4. Univariate analysis revealed FGM and family history of HBV infection were significantly associated with HBV infection among pregnant women (*p* < 0.05). Further analysis using multiple logistic regression revealed FGM (OR *=* 3.08, 95% CI *=* 1.1–8.7, *p*=0.03) significantly influencing the occurrence of HBV infection. The presence of FGM increased the odds of HBV infection by 2.08 times compared with a woman without FGM as shown in Table 4.

## 4. Discussion

STBBIs are a public health burden and disproportionately affect women globally. Intrapregnancy STBBIs are particularly associated with adverse pregnancy outcomes [5]. However, antenatal screening services and treatment for infections such as syphilis and HIV have been proven effective in pregnancy outcomes [12, 13]. Therefore, antenatal screening services for STBBIs, integrated with the provision of syndromic management of infections during pregnancy in antenatal clinics, should be prioritized.

The prevalence of the STBBIs among pregnant women in Jirapa municipality was 11.4%, higher than other prevalence studies conducted in Ghana [14–16] but lower than a prevalence rate of 16.7% in rural Ghana [17], and the national prevalence of 12.3% among pregnant women in the country [18]. This points to increased chances of the transmission of some STBBIs from mother-to-child (MTCT) and maternal complications due to infections. Timely screening, administration of immunoprophylaxis, vaccination, and education on STBBIs are required for expectant mothers.

Within the Sub-Saharan Africa region, the estimated HBV seroprevalence in the present study is lower than in Uganda (11.8%) [19] but higher than in Gambia (9.2%), Nigeria (8.3%), Tanzania (5.2%), Ethiopia (4.7%), and Rwanda (3.1%) [20–24]. The national and regional variations of HBV infection could be explained by the knowledge of HBV and access to vaccines. Additionally, sociocultural and behavioral practices, and geographical variations could be contributory factors [15].

The seroprevalence of HCV (0.8%) in our study is far less compared to a study in Ghana by Ephraim et al. in 2017 who reported a prevalence of 7.7% [16]. However, comparable lower and higher prevalence is being reported in other parts of Africa including 1.7% in Nigeria [22, 25–29]. The risk of MTCT of HCV ranges between 5–15%, complicated by the unavailability of protocols to determine viraemia levels in seropositive mothers. Vertical transmission of HCV is near zero in mothers negative for HCV RNA but 4–15% in HCV RNA-positive mothers [30]. Despite the low prevalence of HCV in this study, the introduction of further testing to determine the viraemia levels of seropositive mothers will help avert MTCT in Ghana.

The 0.8% prevalence of HIV recorded among pregnant women in our study is comparably higher to 0.6% obtained in a rural setting in Ghana [17] but lower compared to the prevalence reported in some urban areas [31, 32]. In southwestern Nigeria, and some parts of Africa, a study among pregnant women revealed higher HIV prevalence [29, 31, 33] but lower in Cuttack, India (0.5%) [34]. With the advances made in antiretroviral therapy, infected mothers can be managed to effectively prevent MTCT and lessen the economic burden due to HIV morbidity and mortality in both mothers and babies.

Syphilis is one of the curable infections in pregnancies but with adverse complications if undiagnosed or untreated. Congenital syphilis is the ramifications of maternal syphilis that is undiagnosed, inadequately treated, or untreated [35]. The 0.8% seroprevalence in our study is lower than reported in similar research works in Ghana, Nigeria, Ethiopia, and Sudan [36–40] but higher than 0.24% obtained in Ho, Ghana, and 0.19% in Portugal [32, 35]. Despite the low prevalence in our study, it still remains a public health concern due to its adverse effects on newborn.

The knowledge of the pregnant women on STBBIs was good with 66.3% cognizant of MTCT of infections. The knowledge of MTCT of infections is comparably higher than a similar study which recorded a 34.7% knowledge level [41]. Health promotion activities of early preventive strategies, healthy lifestyle, and well-being should equally be directed at raising community awareness of syphilis and hepatitis in the study area.

The univariate risk factors associated with and predictive of the occurrences of the STBBIs were FGM and gravidity. Also, a family history of HBV was found associated with HBV infection in the study. Family history of hepatitis [15], history of close contact with hepatitis patients [42], and presence of liver disease in the household [28] have been previously identified as risk factors to STBBIs as corroborated by our study. It is unclear how gravidity is associated with the risks of STBBIs. However, behaviors associated with gravidities such as the account of abortion and numerous sexual exposures have been reported as risk of STBBIs. A striking observation in the study was the occurrence of no STBBIs among pregnant women vaccinated against the hepatitis B virus. Hepatitis B vaccination is reported to prevent clinical infection, and risk of HBV infection in sexually active individuals [43]. A study in China associated low rate of HIV infection in patients vaccinated against HBV [44]. Not all, mothers' completion of the hepatitis B vaccination schedule was protective against infection with HBV during childhood [45]. These findings imply a protective role of HBV vaccination and reduced risk of some STBBIs as we found no infection among participants vaccinated against HBV in this study.

Female genital mutilation (FGM) involves removing flesh from the private part of woman. The national prevalence is estimated to be 11.7% [46]. In the Upper West region where this study was conducted, the prevalence is estimated at between 41.1% and 50.5% [46, 47]. Aside from the adverse effect of FGM on sexual satisfaction, the risk of HBV, HCV, and HIV among FGM victims might be attributed to the greater percentage (84%) of the cases carried out by traditional practitioners locally called “wanzams” [47]. Our study found that 23.0% of pregnant women had experienced FGM. Females with FGM have a lower sexual function index compared to their uncircumcised counterparts [48] resulting in dryness and bruises during sex increasing the risk of blood-borne infections. Other effects that increase the risk of infection include open sores and bleeding [49] associated with FGM. With regards to sexual behavior, circumcised women are less likely to decline sex and demand condom use than uncircumcised women as reported in a study in Kenya [50] which could increase their risk of contracting STBBIs.

## 5. Conclusion

In our study, FGM was a strong risk and predictive factor of STBBIs among pregnant women in Jirapa municipality. Concerted efforts to terminate the practice of FGM in the study area and the augmentation of an integrated cultural approach in the improvement of reproductive health practices are recommended by all stakeholders.

## Figures and Tables

**Table 1:** Sociodemographic, medical, and obstetric characteristics of antenatal attendees in Jirapa municipality, Ghana.

Variables	Frequency (*N* = 246)	%*N*
*Age*		
<20	43	17.5
20–29	118	48.0
30–39	81	32.9
≥40	4	1.6

*Religion*		
Christianity	216	87.8
Islam	27	11.0
Traditional	3	1.2

*Marital status*		
Single	36	14.6
Married	210	85.4

*Educational level*		
No formal education	47	19.1
Primary	116	47.2
Secondary	42	17.0
Tertiary	41	16.7

*Occupation*		
Housewife	128	52.0
Trader	78	31.7
Employed	40	16.3

*Medical history*		
Blood transfusion	25	10.2
Scarification	142	57.7
FGM	57	23.2
Surgical operation	22	8.9
Abortion	34	13.8
Dental care	9	3.7
Family history of HBV infection	9	3.7
Family history of HCV infection	4	1.6
Family history of HIV infection	3	1.2
History of STBBIs	15	6.1
Vaccination against HBV	34	13.8

*Gravidity*		
Primigravida	90	36.6
Multigravida	156	63.4

*Parity*		
Nullipara	97	39.4
Primipara	66	26.8
Multipara	83	33.8

Note. FGM: female genital mutilation.

**Table 2:** Knowledge level of pregnant women attending antenatal clinic on sexually transmitted and blood-borne infections and risk factors in Jirapa municipality, Ghana.

Variables	Frequency (*N* = 246)	Percentage (%*N*)
*Transmission*		
Knowledge of infections transmitted from mother to child	163	66.3
Knowledge of HIV transmission from mother to child	231	93.9
Knowledge of viral hepatitis transmission from mother to child	165	67.1
Knowledge of syphilis transmission from mother to child	132	53.7

*Risk factors of infections*		
Knowledge of HIV risk factors	240	97.6
Knowledge of hepatitis risk factors	183	74.4
Knowledge of syphilis risk factors	187	76.0

*Signs/symptoms*		
Knowledge of signs and symptoms of HIV	221	89.8
Knowledge of signs and symptoms of viral hepatitis	233	94.7
Knowledge of signs and symptoms of syphilis	230	93.5

*Prevention*		
Knowledge on prevention of HIV	243	98.8
Knowledge on prevention of viral hepatitis	243	98.8
Knowledge on prevention of syphilis	241	98.0

**Table 3:** Factors associated and influencing sexually transmitted blood-borne infections among pregnant women attending antenatal clinic in Jirapa municipality, Ghana.

Variables		STBBIs		Chi-square	Logistic regression	
		Negative (%T)	Positive (%T)	*X* ^2^ (*p* value)	AOR (95% CI)	*p* value
Age	≤26	118 (48.0)	15 (6.1)	0.003 (*0.96*)	1	
	>26	100 (40.7)	13 (5.3)		1.02 (0.5–2.3)	*0.96*

Religion	Christian	119 (77.6)	25 (10.2)	**7.32 (0.02)** ^*∗∗*^		
	Muslim	26 (10.6)	1 (0.4)			
	Traditional	1 (0.4)	2 (0.8)			

Marital status	Single	33 (13.4)	3 (1.2)	0.39 *(0.54)*	1	
	Married	185 (75.2)	25 (10.2)		1.49 (0.4–5.2)	*0.54*

Education	Low	140 (56.9)	23 (9.3)	3.57 *(0.07)*	1	
	High	78 (31.7)	5 (2.0)		0.39 (0.1–1.1)	*0.07*

Occupation	Salaried	181 (74.2)	25 (10.2)	0.57 *(0.45)*	1	
	Nonsalaried	35 (14.3)	3 (1.2)		0.62 (0.2–2.2)	*0.46*

FGM	No	173 (70.3)	16 (6.5)	**6.88 (0.009)**	1	
	Yes	45 (18.3)	12 (4.9)		2.88 (1.3–6.5)	**0.01**

Scarification	No	91 (37.0)	13 (5.3)	0.22 *(0.64)*	1	
	Yes	127 (51.6)	15 (6.1)		0.83 (0.4–1.8)	*0.64*

Blood transfusion	No	196 (79.7)	25 (10.2)	0.01 *(0.92)*	1	
	Yes	22 (8.9)	3 (1.2)		1.07 (0.3–3.8)	*0.92*

Abortion	No	188 (76.7)	23 (9.4)	0.42 *(0.52)*	1	
	Yes	29 (11.9)	5 (2.0)		1.41 (0.5–4.0)	*0.52*

Surgical operation	No	197 (80.4)	24 (9.8)	0.13 *(0.72)*	1	
	Yes	20 (8.2)	4 (1.6)		0.76 (0.2–3.4)	*0.72*

STIs	No	207 (84.1)	26 (10.6)	3.70 *(0.05)*	1	
	Yes	11 (4.5)	2 (0.2)		3.13 (0.9–10.6)	*0.07*

Dental surgery	No	208 (84.9)	28 (11.4)	1.20 *(0.27)*^*∗∗*^		
	Yes	9 (3.7)	0 (0.0)			

Family history of HBV	No	212 (86.2)	25 (10.2)	**4.46 (0.04)**	1	
	Yes	6 (2.4)	3 (1.2)		4.24 (1.0–18.0)	*0.05*

Family history of HCV	No	216 (87.8)	26 (10.6)	**6.01 (0.01)** ^*∗∗*^		
	Yes	2 (0.8)	2 (0.8)			

Family history of HIV	No	216 (87.8)	27 (11.0)	1.45 *(0.23)*^*∗∗*^		
	Yes	2 (0.8)	1 (0.4)			

Gravidity	Primigravida	84 (34.3)	5 (2.0)	**4.66 (0.04)**	1	
	Multigravida	133 (54.3)	23 (9.4)		2.91 (1.1–7.9)	**0.04**

Parity	Nullipara	88 (35.9)	8 (3.3)	3.69 *(0.16)*	1	*0.17*
	Primipara	60 (24.5)	6 (2.4)		1.10 (0.4–3.3)	*0.87*
	Multipara	69 (28.2)	14 (5.7)		2.2 (0.9–5.6)	*0.09*

Knowledge	Moderate	42 (17.1)	3 (1.2)	1.2 *(0.27)*	1	
	Good	176 (71.5)	25 (10.2)		1.99 (0.6–6.9)	*0.28*

HBV vaccination	No	184 (74.0)	28 (11.4)	**5.07 (0.024)** ^*∗∗*^		
	Yes	34 (13.8)	0 (0.0)			

Note. FGM: female genital mutilation. ^*∗∗*^Variable and *p* value not considered because a cell value is less than 1 or more than 30% of cells have value less than 5. Bold values are statistically significant at *p* < 0.05.

**Table 4:** Factors associated and influencing HBV infections among pregnant women attending antenatal clinic in Jirapa municipality, Ghana.

Variables		HBV infection		Chi-square	Logistic regression	
		Negative (%T)	Positive (%T)	*X* ^2^ (*p* value)	AOR (95% CI)	*p* value
		*N* = 222	*N* = 24			
Age	≤26	121 (49.2)	12 (4.9)	0.18 (*0.83*)	1	
	>26	101 (41.1)	12 (4.9)		0.81 (0.3–2.6)	*0.72*

Religion	Christian	195 (77.6)	21 (10.2)	*12.16 (0.02)* ^*∗∗*^		
	Muslim	26 (10.6)	1 (0.4)			
	Traditional	1 (0.4)	2 (0.8)			

Marital status	Single	33 (13.4)	3 (1.2)	0.10 *(0.78)*	1	
	Married	189 (76.8)	21 (8.5)		1.1 (0.3–4.8)	*0.88*

Education	Low	144 (58.5)	19 (7.7)	*1.98 (0.18)*	1	
	High	78 (31.7)	5 (2.0)		0.27 (0.1–1.3)	*0.11*

Occupation	Salaried	185 (75.8)	21 (8.6)	0.19 *(0.78)*	1	
	Nonsalaried	35 (14.3)	3 (1.2)		2.1 (0.3–14.9)	*0.45*

FGM	No	175 (71.1)	14 (5.7)	**5.11 (0.04)**	1	
	Yes	47 (19.1)	10 (4.1)		3.08 (1.1–8.7)	**0.03**

Scarification	No	92 (37.4)	12 (4.9)	0.65 *(0.52)*	1	
	Yes	130 (52.8)	12 (4.9)		0.48 (0.2–1.3)	*0.16*

Blood transfusion	No	199 (80.9)	2 (0.8)	0.10 *(1.00)*^*∗∗*^		
	Yes	23 (9.3)	3 (1.2)			

Abortion	No	191 (78.0)	20 (8.2)	0.17 *(0.76)*	1	
	Yes	30 (12.2)	4 (1.6)		1.32 (0.4–4.7)	*0.67*

Surgical operation	No	200 (81.6)	23 (9.4)	0.75 *(0.49)*	1	
	Yes	21 (8.6)	1 (0.4)		0.28 (0.03–2.3)	*0.24*

STIs	No	210 (85.4)	21 (8.5)	*1.90 (0.17)*	1	
	Yes	12 (4.9)	3 (1.2)		3.92 (0.44–34.13)	*0.22*

Dental surgery	No	212 (86.5)	24 (9.8)	1.02 *(0.61)*^*∗∗*^		
	Yes	9 (3.7)	0 (0.0)			

Family history of HBV	No	216 (87.8)	21 (8.5)	**5.90 (0.04)**	1	
	Yes	6 (2.4)	3 (1.2)		2.72 (0.5–15.6)	*0.26*

Family history of HCV	No	220 (89.4)	22 (8.9)	*7.48 (0.05)* ^*∗∗*^		
	Yes	2 (0.8)	2 (0.8)			

Family history of HIV	No	220 (89.4)	23 (9.3)	1.92 *(0.27)*^*∗∗*^		
	Yes	2 (0.8)	1 (0.4)			

Gravidity	Primigravida	84 (34.3)	5 (2.0)	*2.76 (0.12)*	1	
	Multigravida	137 (55.9)	19 (7.8)		2.38 (0.2–36.3)	*0.53*

Parity	Nullipara	90 (36.7)	6 (2.4)	*4.97 (0.08)*	1	
	Primipara	61 (24.9)	5 (2.0)		0.65 (0.5–9.1)	*0.75*
	Multipara	70 (28.6)	13 (5.3)		1.43 (0.1–17.8)	*0.78*

Knowledge	Moderate	43 (17.5)	2 (0.8)	1.77 *(0.27)*	1	
	Good	179 (72.8)	22 (8.9)		2.90 (0.6–136.3)	*0.18*

HBV vaccination	No	188 (76.4)	24 (9.8)	*4.27 (0.05)* ^*∗∗*^		
	Yes	34 (13.8)	0 (0.0)			

Note. FGM: female genital mutilation. ^*∗∗*^Variable and *p* value not considered because a cell value is less than 1 or more than 30% of cells have value less than 5. Bold values are statistically significant at *p* < 0.05.

## Data Availability

The data supporting the current study are available from the corresponding author upon request.

## Additional Points

In low resource settings, a lot of cases of HBV and HCV infections are thought to be through congenital infection. This risk factor could not be assessed in our study. Also, there are many infections classified as STBBIs. However, only HCV, HBV, HIV, and syphilis were studied and cannot be generalized for all STBBIs.
